# MicroRNA-362 induces cell proliferation and apoptosis resistance in gastric cancer by activation of NF-κB signaling

**DOI:** 10.1186/1479-5876-12-33

**Published:** 2014-02-05

**Authors:** Jin-tang Xia, Lian-zhou Chen, Wei-hua Jian, Ke-Bing Wang, Yong-zhen Yang, Wei-ling He, Yu-long He, De Chen, Wen Li

**Affiliations:** 1 Department of General Surgery, The third Affiliated Hospital, Guangzhou Medical University, #63 Duobao Road, Guangzhou, Guangdong 510150, China; 2 Laboratory of General Surgery, The First Affiliated Hospital, Sun Yat-sen University, #58 Zhongshan er Road, Guangzhou, Guangdong 510080, China; 3 Department of General Surgery, Guangzhou First Municipal People’s Hospital, Guangzhou Medical University, Guangzhou, Guangdong 510180, China; 4 Department of Gastrointestinal Surgery, The First Affiliated Hospital, Sun Yat-sen University, Guangzhou, Guangdong 510080, China

**Keywords:** miR-362, NF-κB, CYLD, Gastric cancer, Proliferation, Apoptosis

## Abstract

**Background:**

According to cancer-related microRNA (miRNA) expression microarray research available in public databases, miR-362 expression is elevated in gastric cancer. However, the expression and biological role of miR-362 in gastric progression remain unclear.

**Methods:**

miR-362 expression levels in gastric cancer tissues and cell lines were determined using real-time PCR. The roles of miR-362, in promoting gastric cancer cell proliferation and apoptosis resistance, were assessed by different biological assays, such as colony assay, flow cytometry and TUNEL assay. The effect of miR-362 on NF-κB activation was investigated using the luciferase reporter assay, fluorescent immunostaining.

**Results:**

MiR-362 overexpression induced cell proliferation, colony formation, and resistance to cisplatin-induced apoptosis in BGC-823 and SGC-7901 gastric cancer cells. MiR-362 increased NF-κB activity and relative mRNA expression of NF-κB–regulated genes, and induced nuclear translocation of p65. Expression of the tumor suppressor CYLD was inhibited by miR-362 in gastric cancer cells; miR-362 levels were inversely correlated with CYLD expression in gastric cancer tissue. MiR-362 downregulated CYLD expression by binding its 3′ untranslated region. NF-κB activation was mechanistically associated with siRNA-mediated downregulation of CYLD. MiR-362 inhibitor reversed all the effects of miR-362.

**Conclusion:**

The results suggest that miR-362 plays an important role in repressing the tumor suppressor CYLD and present a novel mechanism of miRNA-mediated NF-κB activation in gastric cancer.

## Background

Gastric cancer is the fourth most common cancer and the second leading cause of cancer death worldwide [1]. Surgery is the main treatment for operable gastric cancer; however, recurrence and metastasis are very common [2,3]. The combination of surgery and chemotherapy has recently emerged as an effective strategy for gastric cancer therapy, improving disease-free survival and reducing the risk of recurrence and metastasis as compared with surgery only in multiple trials [4,5]. However, clinical responses to chemotherapy vary greatly, which leads to different curative effects for gastric cancer patients [6]. Although anti-cancer drugs generally kill tumor cells by inducing apoptosis, recent advances have shown that most solid tumors are generally or particularly resistant to chemotherapy-induced apoptosis [7-9]. Therefore, the chemotherapy drug susceptibility of cancer cells with one or more gene mutations and apoptosis pathway defects directly influences the curative effects.

NF-κB is constitutively elevated in many human tumors, both hematological and solid [10], including gastric cancer [11]. Many studies have shown that activated NF-κB signaling is highly associated with tumorigenesis, tumor progression, and therapy resistance. It plays an important role in oncogenesis due to its anti-apoptosis and pro-proliferation activities [12-15]. Many observations indicate that NF-κB suppresses apoptosis through transcriptional regulation of the expression of anti-apoptotic genes, including *TRAF1*, *TRAF2*, c-*IAP1*, and c-*IAP2*, which blocks caspase-8 activation, and the Bcl-2 homologues A1/Bfl-1, Bcl-xL, IEX-1, and XIAP [12]. Over the years, much progress has been made in the study of the regulatory mechanisms of NF-κB signaling. Ubiquitin modification has been proven to play a crucial role in NF-κB signaling activation [16-18]. Conversely, ubiquitin deconjugation mediated by deubiquitinases such as CYLD negatively regulates NF-κB signaling [19,20]. CYLD abrogates the activation of NF-κB signaling via its deubiquitinating activity on multiple NF-κB signaling mediators, including TRAF2, TRAF6, RIP1, TAK1, NEMO, and BCL3 [21-23]. Furthermore, multiple studies have demonstrated that CYLD is a tumor suppressor associated with the inhibition of cell proliferation and induction of apoptosis [24,25]. In hepatocellular carcinoma cells, CYLD downregulation leads to apoptosis resistance [26].

It has been demonstrated that aberrant microRNA (miRNA) expression is associated with various diseases and cancers [27]. Recent evidence revealed that miRNA expression significantly correlates with the progression and prognosis of gastric cancer [28]. In gastric cancer patients, upregulated miR-20b, miR-142-5p, miR-150, and miR-375, and decreased miR-124a, miR-125a-5p, miR-146a, and miR-45 were associated with shorter survival times [29]. Several miRNAs appear to predict or affect the response to chemotherapy. MiR-15b or miR-16 overexpression increases gastric cancer cell sensitivity to vincristine, whereas miR-15b or miR-16 downregulation increases gastric cell sensitivity to related drugs [30].

From public databases and datasets on gastric cancer–related miRNA expression microarray, we found that miR-362 is upregulated in gastric cancer. Though miR-362 was reported to be upregulated in acral melanomas as compared to non-acral melanomas [31], the function and mechanism of miR-362 in gastic cancer remains unknown. In the present study, we found that miR-362 was significantly associated with cell proliferation and apoptosis resistance of gastric cancer. Moreover, miR-362 activated NF-κB signaling through directly targeting of the 3′ untranslated region (3′-UTR) and suppression of CYLD in human gastric cancer cells. Thus, our results suggest that miR-362 might play an important role in promoting the development and progression of gastric cancer.

## Materials and methods

### Cell culture

Primary normal human gastric epithelial cells (NGEC) were established from gastric biopsy specimens obtained from upper gastrointestinal endoscopy and cultured as described previously [32]. The gastric cancer cell lines SGC-7901, BGC-823, HGC-27, MKN-28, and MGC-803 were maintained in DMEM (Invitrogen, Carlsbad, CA, USA) supplemented with 10% fetal bovine serum (HyClone, Logan, UT, USA).

### Tissue specimens

Ten paired gastric tumor and adjacent non-tumor tissues, and another 10 freshly collected gastric cancer tissues were collected and histopathologically diagnosed at the Departments of Gastrointestinal Surgery and Pathology, The First Affiliated Hospital, Sun Yat-sen University. Patient consent and Institutional Research Ethics Committee approval were obtained prior to the use of these clinical materials for research purposes.

### Plasmids, siRNA, and transfection

The gene for human miR-362 was PCR-amplified from genomic DNA and cloned into a pMSCV-puro retroviral vector (Clontech, Mountain View, CA). The primers used were as follows: miR-362-up, 5′-GCCAGATCTACATGCTTGGTCCCTACCC-3′ and miR-362-dn, 5′-GCCCTCGAGCAGGTGCTGGATGTATTTGG-3′. The region of human CYLD 3′-UTR, generated by PCR amplification of SGC-7901 cell DNA, was cloned into pEGFP-C1 (Clontech, Mountain View, CA, USA) and pGL3 vectors (Promega, Madison, WI, USA). The primers used (forward and reverse) were as follows: CYLD-3′UTR-GFP-up, 5′-GCCCTCGAGCTTGACTCCGTTCCCCTTCAGAC-3′; CYLD-3′UTR-GFP-dn, 5′-GCCGGATCCAACCAAGGGCAGTTGAGTC-3′ and CYLD-3′UTR-luc-up, 5′-GCCCCGCGGCTCCGTTCCCCTTCAGAC-3′; CYLD-3′UTR-luc-dn, 5′-GCCCTGCAGAACCAAGGGCAGTTGAGTC-3′. The siRNAs used were CYLD siRNA#1: 5′-GUACCGAAGGGAAGUAUAGUU-3′ and CYLD siRNA#2: 5′-CGCGCUGUAACUCUUUAGCAUU-3′. MiR-362 inhibitor and negative control were purchased from RiboBio (Guangzhou, Guangdong, China). Plasmid and siRNA transfection were performed using Lipofectamine 2000 (Invitrogen) according to the manufacturer’s instructions.

### Western blotting

Western blotting was performed according to standard methods as previously described [33] using anti-p65, anti-p84, anti-GFP (Cell Signaling, Danvers, MA, USA), and anti-CYLD antibodies (Abcam, Cambridge, MA, USA). The membranes were stripped and reprobed with anti–α-tubulin antibody (Sigma-Aldrich, Saint Louis, MO, USA) as a loading control.

### RNA extraction and real-time quantitative PCR

Total miRNA from cultured cells and freshly collected gastric tissues was extracted using a mirVana miRNA Isolation Kit (Ambion, Austin, TX, USA) according to the manufacturer’s instructions. cDNA was synthesized from 10 ng total RNA using a TaqMan miRNA Reverse Transcription Kit (Applied Biosystems, Foster City, CA, USA); Expression levels of miR-362 were quantified using a miRNA-specific TaqMan MiRNA Assay Kit (Applied Biosystems). MiRNA expression was defined based on the threshold cycle (Ct); relative expression levels were derived using 2^-[(Ct of miR-362) – (Ct of U6)]^ after normalization to reference U6 small nuclear RNA expression.

Total RNA was extracted from cells using TRIzol (Invitrogen) according to the manufacturer’s instructions. RNA (2 μg) from each sample was used for cDNA synthesis primed with random hexamers. The primers (forward and reverse) used for gene expression were: CCND1, 5′-TCCTCTCCAAAATGCCAGAG-3′ and 5′-GGCGGATTGGAAATGAACTT-3′; MYC, 5′-TCAAGAGGCGAACACACAAC -3′ and 5′-GGCCTTTTCATTGTTTTCCA-3′; BCL2L1, 5′-TTCAGTGACCTGACATCCCA-3′ and 5′-CTGCTGCATTGTTCCCATAG-3′; FLIP, 5′-TTTCTTTGCCTCCATCTTGG-3′ and 5′-GGGGGAGTTCGTCCTGTC-3′; XIAP, 5′-GACCCTCCCCTTGGACC-3′ and 5′-CTGTTAAAAGTCATCTTCTCTTGAAA-3′; TNF, 5′-CCAGGCAGTCAGATCATCTTCTC-3′ and 5′-AGCTGGTTATCTCTCAGCTCCAC-3′; IL-8, 5′-TGCCAAGGAGTGCTAAAG-3′ and 5′-CTCCACAACCCTCTGCAC-3′; COX-2, 5′-GGCGCTCAGCCATACAG-3′ and 5′-CCGGGTACAATCGCACTTAT-3′. Expression data were normalized to the geometric mean of the housekeeping gene *GAPDH* (forward and reverse primers: 5′-GACTCATGACCACAGTCCATGC-3′ and 5′-AGAGGCAGGGATGATGTTCTG-3′) to control expression level variability and were derived using 2-^[(Ct of gene) – (Ct of GAPDH)]^, where Ct represents the threshold cycle for each transcript.

### MTT assay

Cells (2000) were seeded in 96-well plates and stained at the indicated time points with 100 μL sterile MTT dye (0.5 mg/mL, Sigma-Aldrich) for 4 h at 37°C. The culture medium was removed and 150 μL DMSO (Sigma-Aldrich) was added. Absorbance was measured at 570 nm, with 655 nm as the reference wavelength. All experiments were performed in triplicate.

### Colony formation assay

Cells (1000) were plated in 6-well plates and cultured for 10 days. Colonies were fixed with 10% formaldehyde for 5 min and stained with 1.0% crystal violet for 30 s.

### Flow cytometry analysis

Cells were harvested by trypsinization, washed in ice-cold PBS, and fixed in 80% ice-cold ethanol in PBS. Before staining, cells were pelleted using a chilled centrifuge and resuspended in cold PBS. Bovine pancreatic RNase (Sigma-Aldrich) was added to a final concentration of 2 μg/mL and cells were incubated at 37°C for 30 min, followed by incubation with 20 μg/mL propidium iodide (PI, Sigma-Aldrich) for 20 min at room temperature. The cell cycle profiles of 5 × 10^4^ cells were analyzed using a FACSCalibur flow cytometer (BD, Bedford, MA).

### TUNEL assay

Apoptotic DNA fragmentation was examined using an *in situ* DeadEnd™ Fluorometric Terminal Deoxynucleotidyl Transferase–Mediated dUTP Nick-End Labeling (TUNEL) System Assay Kit (Promega) according to the manufacturer’s protocol. Briefly, 1 × 10^5^ cells/well were plated in 24-well flat-bottom plates and treated with 20 μM cisplatin for 36 h. Cells were fixed in 4% paraformaldehyde at 4°C for 30 min, permeabilized in 0.1% Triton X-100, and labeled with fluorescein-12-dUTP using terminal deoxynucleotidyl transferase. The localized green fluorescence of apoptotic cells (fluorescein-12-dUTP) was detected by fluorescence microscopy (Zeiss Axiovert 100 M, Carl Zeiss, Germany).

### Luciferase assay

Cells (4 × 10^4^) were seeded in triplicate in 24-well plates and cultured for 24 h. NF-κB reporter luciferase plasmid (100 ng), pGL3-CYLD-3′UTR (wt/mut), or control luciferase plasmid, plus 5 ng pRL-TK *Renilla* plasmid (Promega) was transfected into the cells using Lipofectamine 2000 (Invitrogen) according to the manufacturer’s recommendations. Luciferase and *Renilla* signals were measured 36 h after transfection using a Dual Luciferase Reporter Assay Kit (Promega) according to the manufacturer’s protocol.

### Nuclear/cytoplasmic fractionation

Cells were washed with cold PBS and resuspended in buffer containing 10 mM HEPES (pH 7.8), 10 mM KCl, 0.1 mM EDTA, 1 mM Na_3_VO_4_, 1 mM DTT, 1:500 protease inhibitors (Sigma-Aldrich), and 0.2 mM PMSF and incubated on ice for 15 min. Detergent was added and cells were vortexed for 10 s at the highest setting. Nuclei were separated by centrifugation at 4°C, resuspended in buffer containing 20 mM HEPES (pH 7.8), 0.4 M NaCl, 1 mM EDTA, 1 mM Na_3_VO_4_, 1 mM DTT, and 1:500 protease inhibitors, and incubated on ice for 15 min. Nuclear extracts were collected by centrifugation at 14,000 × *g* for 10 min at 4°C.

### Annexin V binding assay

An ApopNexin™ FITC Apoptosis Detection Kit (Millipore, Lake Placid, NY, USA) was used to detect apoptotic cells according to the manufacturer’s instructions. Cells (3 × 10^5^) were seeded in 6-well plates in triplicate and incubated with 20 μM cisplatin or vehicle for 24 hours. Adherent and floating cells were combined, followed by washing with PBS and then with annexin V binding solution. Subsequently, 150 μL annexin V antibody in binding buffer was added to each well and incubated for 15 min, followed by the addition of 1.5 μL 1 mg/mL PI and further incubation for 5 min. Cells (10,000) were analyzed using a FACSCalibur flow cytometer (BD Biosciences). The data were analyzed with CellQuest software to differentiate apoptotic cells (annexin V–positive and PI-negative) from necrotic cells (including late apoptotic cells).

### Statistical analysis

A two-tailed Student’s *t*-test was used to evaluate the significance of the differences between two groups of data in all pertinent experiments; *P* < 0.05 was considered significant.

## Results

### MiR-362 was upregulated in human gastric cancer cell lines and tissues

**Figure 1:**
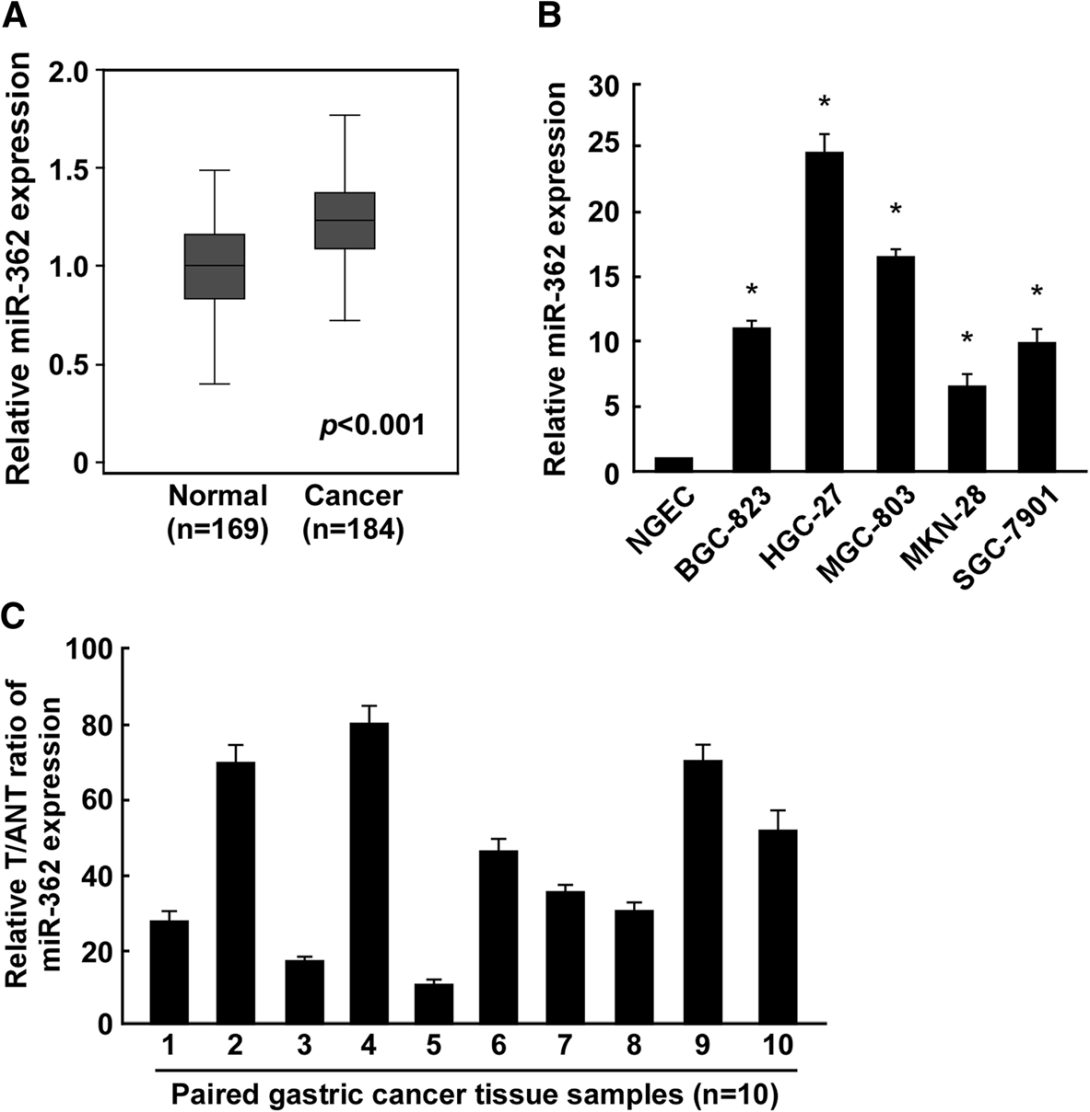
**MiR-362 upregulation in human gastric cancer cell lines and tissues. (A)** High-throughput microarray analysis of published data showing that miR-362 was elevated in gastric cancer tissues (n = 184) as compared with that in noncancerous gastric tissue (n = 169). **(B)** Real-time PCR analysis of miR-362 expression in NGEC and gastric cancer BGC-823, HGC-27, MGC-803, MKN-28, and SGC-7901 cell lines. Transcript levels were normalized to *U6* expression. **(C)** MiR-362 expression in primary gastric cancer tissues (T) with paired adjacent normal tissues (ANT) from 10 patients. Transcript levels were normalized to *U6* expression. Experiments B and C were repeated at least three times. Bars denote the mean of three independent experiments. *P < 0.05.

To identify miRNAs that may be involved in gastric cancer progression, we analyzed a published microarray-based, high-throughput miRNA expression dataset (E-TABM-341, ArrayExpress). We found that miR-362 expression was significantly upregulated in human gastric cancer tissues (n = 184) than that in normal gastric tissues (n = 169) (*P* < 0.001, Figure 1A). Real-time PCR analysis showed marked upregulation of miR-362 expression in all five gastric cancer cell lines as compared with that in NGEC (Figure 1B). Comparative analysis indicated that miR-362 was increased in all 10 gastric tumor tissue specimens as compared with adjacent non-cancerous tissue specimens (Figure 1C). Taken together, these results demonstrate that miR-362 is upregulated in human gastric cancer.

### MiR-362 upregulation promoted cell proliferation and induced apoptosis resistance in gastric cancer

**Figure 2:**
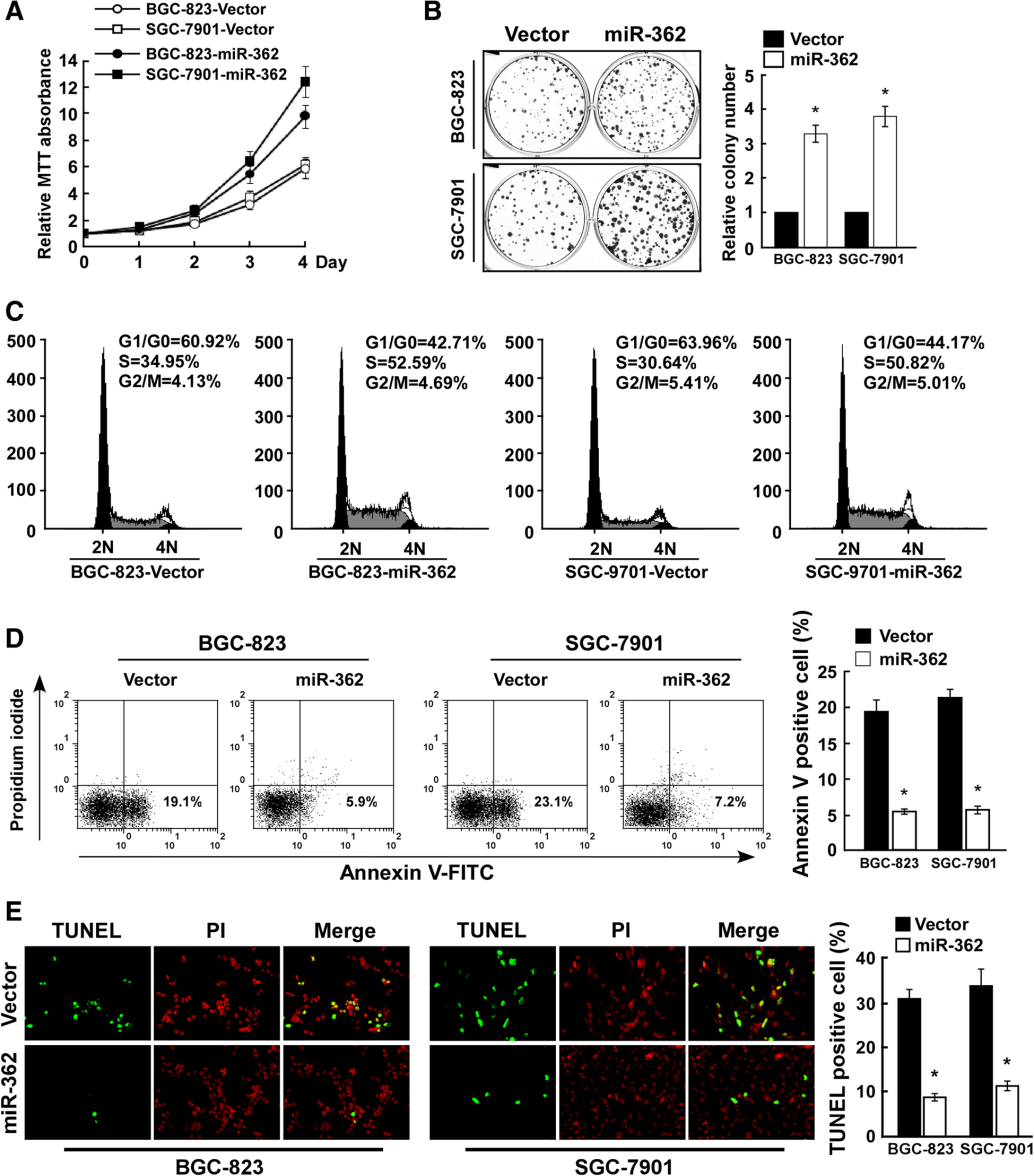
**MiR-362 upregulation promotes cell proliferation and induces apoptosis resistance in gastric cancer cells. (A)** MTT assay revealing that miR-362 upregulation induced growth in BGC-823 and SGC-7901 gastric cancer cells. **(B)** Representative micrographs (left) and quantification (right) of crystal violet–stained cell colonies. **(C)** Flow cytometric analysis of gastric cancer cells transduced with vector or miR-362. **(D)** Annexin V–FITC/PI staining of cells treated with 20 μM cisplatin for 24 h. **(E)** Representative micrographs (left) and quantification of TUNEL-positive cells in cells treated with 20 μM cisplatin for 36 h. Bars denote the mean of three independent experiments. *P < 0.05.

To investigate the biological effect of miR-362 upregulation on gastric cancer progression, the BGC-823 and SGC-7901 gastric cancer cell lines were used to stably express miR-362. MTT assay showed that miR-362 upregulation significantly increased the rate of cell proliferation (Figure 2A), and this was confirmed by colony formation assay (Figure 2B). Flow cytometry revealed a dramatic increase in the percentage of S-phase cells in miR-362–overexpressing BGC-823 (52.59%) and SGC-7901 cells (50.82%) as compared with control BGC-823 (34.95%) and SGC-7901 cells (30.64%), respectively (Figure 2C). Annexin V and TUNEL staining (Figure 2D and 2E) demonstrated that miR-362 overexpression augmented the resistance of gastric cancer cells to apoptosis induced by the cisplatin treatment. These results suggest that miR-362 plays an oncogenic role in gastric cancer cells *in vitro*.

### MiR-362 inhibition reduced cell proliferation and induced apoptosis in human gastric cancer

**Figure 3:**
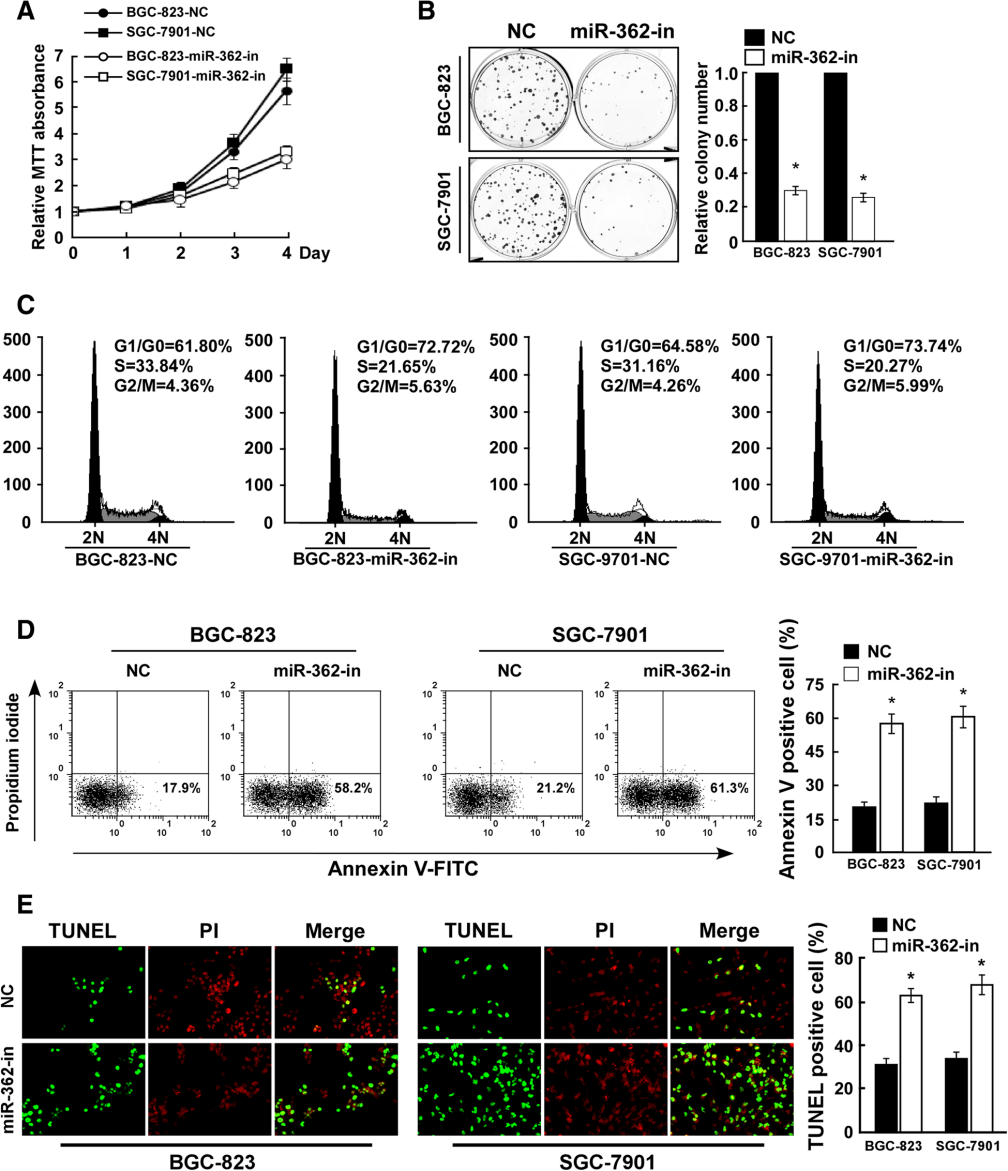
**MiR-362 inhibition suppresses cell proliferation and induces apoptosis of gastric cancer cells. (A)** MTT assay revealing that miR-362 inhibition suppressed the growth of BGC-823 and SGC-7901 gastric cancer cells. **(B)** Representative micrographs (left) and quantification (right) of crystal violet–stained cell colonies. **(C)** Flow cytometric analysis of gastric cancer cells transduced with negative control (NC) or miR-362 inhibitor (miR-362-in). **(D)** Annexin V–FITC/PI staining of cells treated with 20 μM cisplatin for 24 h. **(E)** Representative micrographs (left) and quantification of TUNEL-positive cells in cells treated with 20 μM cisplatin for 36 h. Bars denote the mean of three independent experiments. *P < 0.05.

We examined the effect of miR-362 inhibition on gastric cancer progression. Consistent with the above results, the MTT and colony formation assays showed that miR-362 suppression dramatically inhibited the growth rate of both BGC-823 and SGC-7901 cells as compared with that of control cells (Figure 3A and 3B). Flow cytometry showed that miR-362 inhibition decreased the percentage of cells in S-phase peak but increased that of G1/G0-phase cells (Figure 3C), suggesting that miR-362 inhibition results in G1/S arrest in gastric cancer cells. Annexin V and TUNEL staining demonstrated that miR-362 inhibition decreased resistance to apoptosis in cisplatin-treated gastric cancer cells (Figure 3D and 3E).

### MiR-362 activated the NF-κB pathway

**Figure 4:**
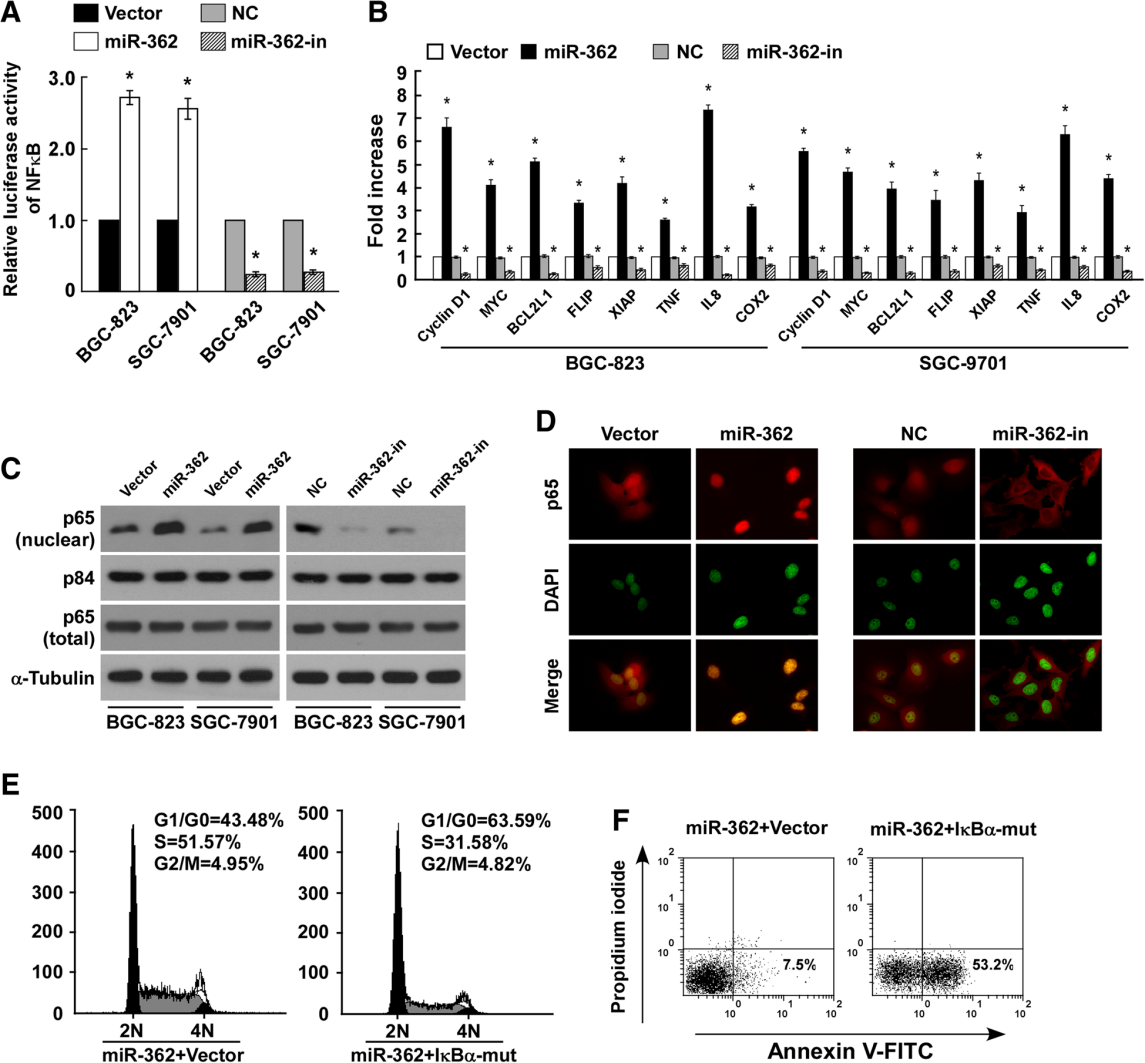
**MiR-362 activates the NF-κB signaling pathway. (A)** NF-κB luciferase reporter activities were analyzed in cells in which miR-362 had been transfected or inhibited (miR-362-in). **(B)** Relative mRNA expression of NF-κB–regulated genes in cells. *GAPDH* was used as a loading control. **(C)** Western blotting of total and nuclear expression of p65. α-Tubulin and the nuclear protein p84 served as loading markers. **(D)** Immunofluorescence staining of subcellular localization of NF-κB p65 in cells. **(E)** Annexin V–FITC/PI staining of BGC-823 cells transfected with miR-362 or miR-362 plus IκBα dominant-negative mutant (IκBα-mut) and treated with 20 μM cisplatin for 24 h. **(F)** Flow cytometric analysis of BGC-823 cells transfected with miR-362 or miR-362 plus IκBα-mut. Bars denote the mean of three independent experiments. *P < 0.05. NC, Negative control.

We investigated the underlying molecular mechanism that might be responsible for the oncogenic roles of miR-362. As the NF-κB signaling pathway is frequently found hyperactivated in gastric tumors [11,34,35], and activation of NF-κB signaling induces cell proliferation and apoptosis resistance [36], we investigated whether miR-362 regulated NF-κB activity. NF-κB reporter luciferase activity and the expression levels of the eight NF-κB target genes were significantly increased in miR-362–overexpressing cells, but were decreased in cells in which miR-362 had been inhibited (Figure 4A and 4B). Though miR-362 had no effect on the total NF-κB/p65 protein expression, cellular fractionation and immunofluorescence staining showed that miR-362 overexpression promoted nuclear accumulation of NF-κB/p65, while miR-362 inhibition reduced nuclear NF-κB/p65 expression (Figure 4C and 4D), indicating that miR-362 activates the NF-κB pathway through promotion of nuclear NF-κB accumulation. Inhibition of NF-κB signaling by the transfection of an IκBα dominant-negative mutant led to a dramatic decrease in S-phase peak cells but increased the G0/G1-phase peak population (Figure 4E) and cisplatin sensitivity in miR-362–overexpressing cells (Figure 4F), suggesting that NF-κB pathway activation is functionally relevant to miR-362–mediated proliferation and anti-apoptosis.

### MiR-362 targeted CYLD directly

**Figure 5:**
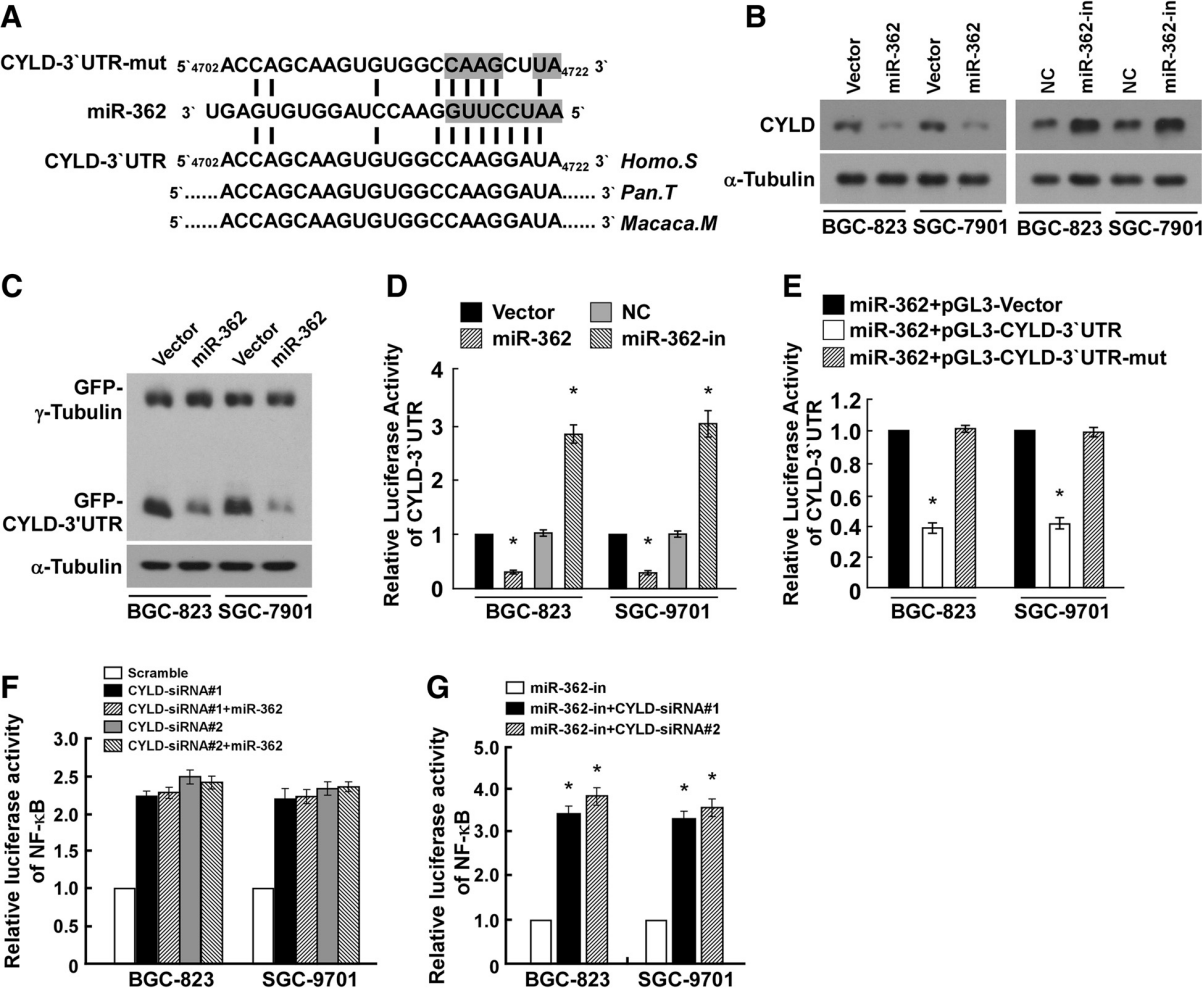
**MiR-362 suppresses CYLD expression by directly targeting the CYLD 3′-UTR. (A)** Predicted miR-362 target sequence in the 3′-UTR of CYLD (CYLD -3′UTR) and mutant containing two mutated nucleotides in the CYLD 3′-UTR (CYLD-3′UTR-mut). **(B)** Western blot of CYLD expression in cells into which miR-362 had been transduced or inhibited (miR-362-in). α-Tubulin served as the loading control. **(C)** Western blot of GFP expression in cells. α-Tubulin served as the loading control. **(D)** Luciferase assay of pGL3-CYLD-3′UTR reporter in cells into which miR-362 had been transduced or inhibited. **(E)** Luciferase assay of cells transfected with pGL3-CYLD-3′UTR or pGL3-CYLD-3′UTR-mut reporter. **(F)** Luciferase reported NF-κB activity in cells transfected with Scramble, CYLD siRNA(s), or CYLD siRNA(s) plus miR-362. **(G)** Luciferase reported NF-κB activity in cells transfected with miR-362 inhibitor or miR-362 inhibitor plus CYLD siRNA(s). Bars denote the mean ± SD of three independent experiments. *P < 0.05. NC, Negative control.

CYLD deubiquitinase is a key negative regulator of NF-κB signaling [21-23]. Analysis using publicly available algorithms (TargetScan, PicTar, miRanda) showed that CYLD is a potential target of miR-362 (Figure 5A). Western blotting analysis revealed that CYLD expression was dramatically repressed by miR-362 overexpression, or induced by miR-362 inhibition (Figure 5B). To examine whether miR-362–induced CYLD downregulation was mediated by the CYLD 3′-UTR, we subcloned the CYLD 3′-UTR fragment containing the miR-362 binding site into pEGFP-C1 and pGL3 dual luciferase reporter vectors. MiR-362 overexpression only decreased the expression of the GFP vector containing the CYLD 3′-UTR (Figure 5C), but had no effect on GFP–γ-tubulin expression, suggesting that miR-362 specifically affected the CYLD 3′-UTR. Reduced luciferase activity was observed following miR-362 overexpression in both BGC-823 and SGC-7901 cells, whereas the repressive effect of miR-362 on luciferase activity of the CYLD 3′-UTR was abolished by the miR-362 inhibitor (Figure 5D). MiR-362 overexpression had no effect on the luciferase activity of CYLD-3′UTR-mut, which contained point mutations in the miR-362–binding seed region of the CYLD 3′-UTR (Figure 5E). Collectively, our results demonstrate that CYLD is a *bona fide* target of miR-362.

### CYLD downregulation is critical for miR-362–mediated NF-κB activation

To further investigate the role of CYLD repression in miR-362–mediated NF-κB activation, we examined the effects of CYLD downregulation on NF-κB activation in BGC-823 and SGC-7901 cells. As expected, CYLD knockdown by the two CYLD-specific siRNAs significantly increased NF-κB reporter luciferase activity and the expression levels of the eight NF-κB target genes (Figure 5F and Additional file 1: Figure S1A). However, further miR-362 overexpression in the CYLD-silenced cells did not have a significant additive effect on NF-κB reporter luciferase activity nor NF-κB target genes expression (Figure 5F and Additional file 1: Figure S1A). Importantly, CYLD downregulation abolished the miR-362 inhibition that induced repression of NF-κB activity and target gene expression (Figure 5G and Additional file 1: Figure S1B). Overall, our results demonstrate that CYLD plays an important role in miR-362–mediated NF-κB activation.

### Clinical correlation between miR-362, CYLD expression, and NF-κB activation in gastric cancer tissues

**Figure 6:**
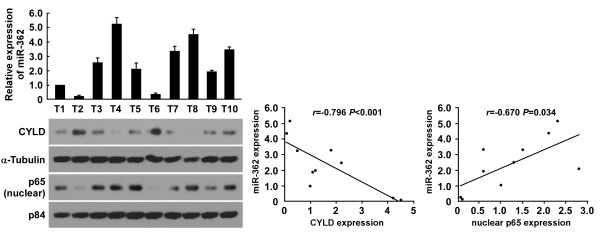
**MiR-362 expression inversely correlates with CYLD expression in gastric cancer tissues.** Analysis (left) and correlation (right) between miR-362 expression and CYLD and nuclear p65 expression levels in 10 freshly collected human gastric cancer tissue specimens. α-Tubulin and the nuclear protein p84 were used as loading controls. Bars denote the mean ± SD of three independent experiments.

We investigated whether the miR-362–induced CYLD repression and NF-κB activation were clinically relevant. MiR-362 levels in the 10 freshly collected gastric cancer specimens were inversely correlated with CYLD expression levels (*r* = -0.796, *P* < 0.001; Figure 6) but positively correlated with nuclear p65 expression (*r* = 0.670, *P* = 0.034). Altogether, our results suggest that miR-362 upregulation activates NF-κB signaling by repressing CYLD, consequently leading to cell proliferation and apoptosis resistance in gastric cancer.

## Discussion

MiRNAs are small noncoding RNAs that regulate the expression of a large number of intracellular target genes. Overexpression of certain miRNAs are important in the regulation of cell proliferation, apoptosis, and differentiation in gastric cancer [37-39]. In the present study, miR-362 expression was upregulated in gastric cancer tissues and cell lines. This is the first study to report that miR-362 overexpression or inhibition with lentivirus vector in BGC-823 and SGC-7901 cells regulated NF-κB activity, p65 protein level, and expression of the NF-κB–related target genes *CCND1*, *MYC*, *BCL2L1*, *FLIP*, *XIPA*, *TNF*, *IL-8*, and *COX-2*. Luciferase assay confirmed that miR-362 directly binds the 3′-UTR of *CYLD* mRNA and inhibits CYLD translation in gastric cancer cells.

The tumor suppressor CYLD is downregulated in many types of cancer, including gliomas, basal cell carcinoma, melanoma, T-cell leukemia, and colon and hepatocellular carcinomas [20,40-43]. Several mechanisms have been proposed to mediate CYLD downregulation in cancers. In skin cancers such as basal cell carcinoma and melanoma, CYLD was repressed at the transcriptional level by the activation of Snail [40,41]. Conversely, CYLD expression in T-cell leukemia was regulated by transcriptional repression by Hes1 [42]. Importantly, a recent study reported that CYLD is a direct target of miR-182, the increased expression of which resulted in CYLD reduction and sustained NF-κB activation in gliomas [20]. In the present study, miR-362 directly targeted CYLD and led to cell proliferation and apoptosis resistance, which we believe is a novel mechanism for reducing CYLD in gastric cancer.

It is widely reported that NF-κB activation is associated with gastric chronic inflammation and gastric cancer [44-46]. NF-κB activation is required for IL-8 release and COX-2 activation, both of which induce the expression of plasminogen activator inhibitor 2 in inflammation caused by *Helicobacter pylori* infection [44]. In gastric cancer, plumbagin inhibits cell growth and enhances apoptosis through suppression of the NF-κB pathway [34]. Furthermore, miR-372 promotes cell growth and inhibits apoptosis through TNFAIP1 downregulation and inhibition of the NF-κB pathway [46]. However, the mechanism of NF-κB activation in gastric cancer remains unclear. In the present study, miR-362 directly targeted the *CYLD* mRNA 3′-UTR and inhibited CYLD translation. The reduction of CYLD ultimately resulted in NF-κB activation. Moreover, as CYLD can be transcriptionally induced by the NF-κB pathway in a negative feedback pathway [47], we may have uncovered a mechanism that leads to persistent NF-κB activation in gastric cancer.

Over the years, adjuvant and neoadjuvant chemotherapy have been taken into account in the treatment strategy for gastric cancer [4,5]. However, the curative effects of chemotherapy in gastric cancer patients are debatable, due to the loss of sensitivity to chemo-induced apopotosis [6]. There is an urgent need to identify an effective parameter that can predict the response to chemotherapy and assist the establishment of individualized therapeutic strategies for gastric cancer patients. Our results suggest that miR-362 overexpression in gastric cancer enhanced cell proliferation and resistance to cisplatin-induced apoptosis in gastric cancer cells. This suggests that miR-362 levels may affect a patient’s sensitivity to chemotherapy. MiR-362 may serve as a predictive factor of patient response towards chemotherapy and may aid in the selection of the optimal therapeutic strategy for gastric cancer patients.

In the present study, miR-362 inhibition decreased cell proliferation, induced apoptosis, and decreased nuclear translocation of p65. This suggests that miR-362 activates the NF-κB pathway without any feedback effect, resulting in persistent NF-κB activation. Although recent discoveries have noted the important roles of many miRNAs in carcinogenesis and cancer progress, data on how miR-362 functions and how it is regulated are scant. In the present study, we identified a very important relationship between miR-362 and NF-κB. As an upstream regulator of the NF-κB pathway, miR-362 downregulation may play an important role in NF-κB pathway suppression.

It was reported that blocking the NF-κB pathway using an IκBα super-repressor such as TNF-α enhances the susceptibility of cells to apoptosis [48]. NF-κB inhibitors enhance the chemotherapeutic sensitivity of colon cancer cells [49]. However, an IκB inhibitor could not block the NF-κB pathway for a prolonged period [48]. Lack of specificity and potential side effects are the major issues in NF-κB inhibitor treatment strategies [50]. Our study presents a new possibility for improving the prognosis of gastric cancer patients with the therapeutic effects of miR-362 inhibition through CYLD downregulation and persistent decrease of NF-κB activity.

## Abbreviations

CYLD: Cylindromatosis; NF-κB: Nuclear factor κB; Bcl2l1: BCL2-like 1; Cox-2: Cytochrome c oxidase subunit II; IL-8: Interleukin 8; TNF: Tumor necrosis factor; XIAP: X-linked inhibitor of apoptosis; FLIP: FLICE-inhibitory protein.

## Competing interests

The authors have declared that no competing interest exists.

## Authors’ contributions

JTX, LZC and WHJ conducted construction of plasmids, cell culture and Western blotting analyses,Real-time PCR analysis and Luciferase assay experiments. KBW, YZY and WLH conducted Flow cytometry analysis, TUNEL assay and immunofluorescence. YLH, WL and DC supervised the project and carried out experimental design and participated in analysis of experiments data. All authors read and approved the final manuscript.

## Supplementary Material

Additional file 1 CYLD plays an important role in miR-362-mediated NF-κB activaton. Click here for file
