# Supplementary material for: MicroRNA-362 induces cell proliferation and apoptosis resistance in gastric cancer by activation of NF-κB signaling
Source: J Transl Med. 2014 Feb 5;12:33. doi: 10.1186/1479-5876-12-33 (PMC3916099; doi:10.1186/1479-5876-12-33)
Supplement: Additional file 1 — CYLD plays an important role in miR-362-mediated NF-κB activaton. [file 1479-5876-12-33-S1.pdf]

## Supplementary Figure 1

A

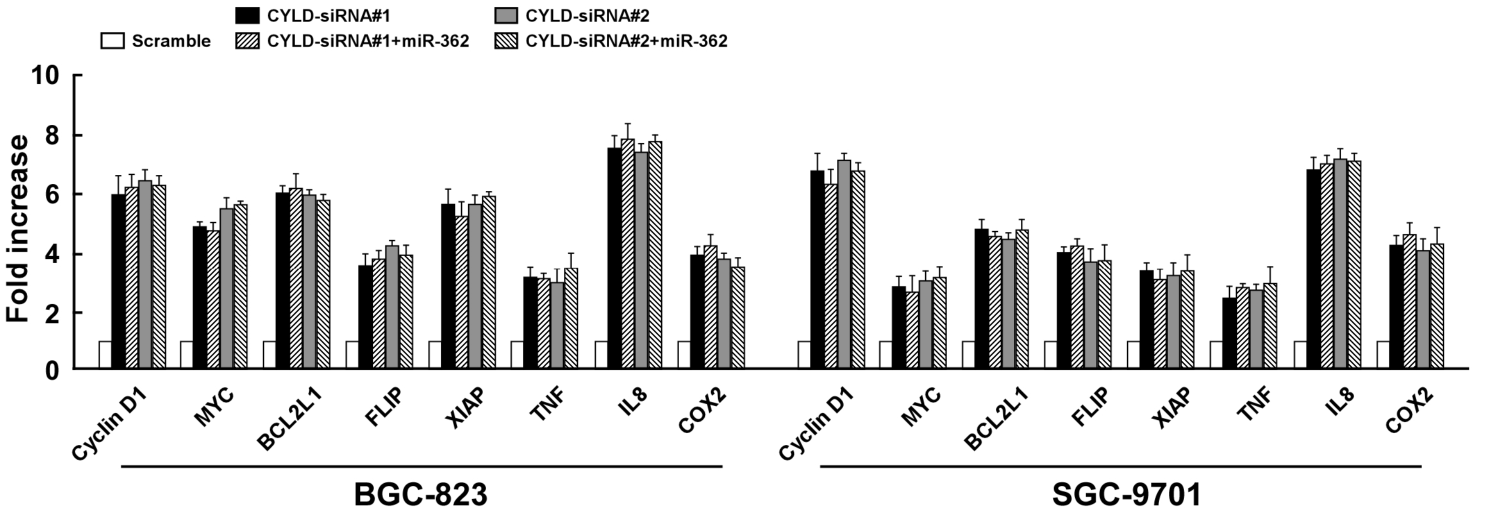


B

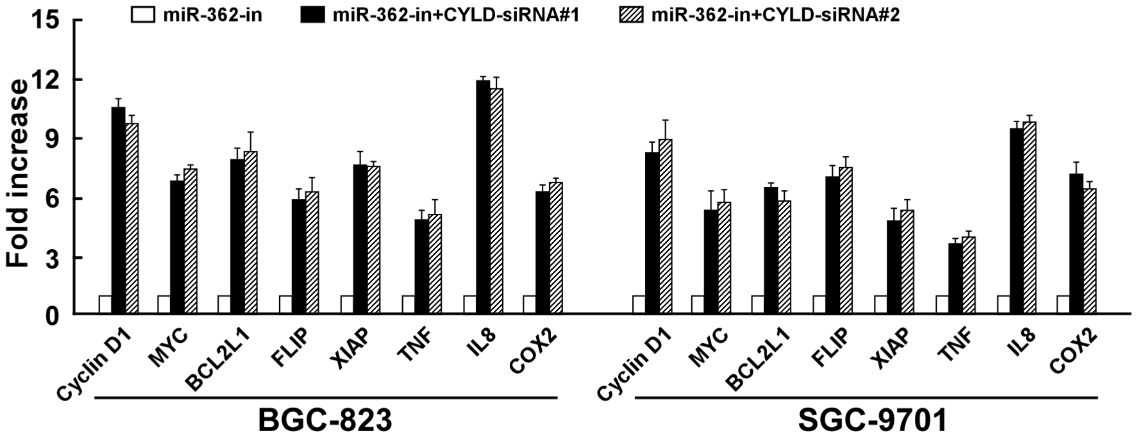


**Supplementary Figure 1. CYLD plays an important role in miR-362-mediated NF-κB activation.**

**(A) Real-time PCR examined the expression of the eight NF-κB target genes in cells transfected with Scramble, CYLD siRNA(s), or CYLD siRNA(s) plus miR-362.**

**(B) Real-time PCR examined the expression of the eight NF-κB target genes in cells transfected with miR-362 inhibitor or miR-362 inhibitor plus CYLD siRNA(s).**

**Bars denote the mean ± SD of three independent experiments. *P<0.05. NC, Negative control.**
